# Efficacy and Safety of Implantable Cardioverter-Defibrillator Use in Peripartum Cardiomyopathy

**DOI:** 10.1016/j.jacadv.2025.101827

**Published:** 2025-05-30

**Authors:** Heather Wheat, Andrew B. Hughey, Angelina Noll, Auras R. Atreya, Thomas Crawford, Jasneet K. Devgun, Michael Ghannam, Madeline K. Mahowald, Josh Errickson, Melinda B. Davis

**Affiliations:** a Division of Cardiovascular Medicine, Department of Internal Medicine, University of Michigan, Michigan, USA; b Department of Electrophysiology, Trinity Health System, Ann Arbor, Michigan, USA; c University of Michigan Medical School, Ann Arbor, Michigan, USA; d Division of Cardiology, University of Louisville School of Medicine, Kentucky, USA; e Section of Cardiac Electrophysiology, Division of Cardiovascular Medicine, Department of Internal Medicine, University of Michigan, Michigan, USA; f Division of Cardiology, Department of Internal Medicine, University of Florida, Jacksonville, Florida, USA; g Consulting for Statistics, Computing & Analytics Research (CSCAR), University of Michigan, Michigan, USA; h Department of Obstetrics and Gynecology, University of Michigan, Michigan, USA

**Keywords:** heart failure, ICD, outcomes, peripartum cardiomyopathy

## Abstract

**Background:**

Implantable cardioverter-defibrillators (ICDs) are recommended in various forms of heart failure, but little is known about outcomes in peripartum cardiomyopathy (PPCM).

**Objectives:**

The authors compared long-term ICD-related outcomes in patients with PPCM vs non-PPCM nonischemic cardiomyopathy (NICM).

**Methods:**

Patients with PPCM and a control group of ethnicity-matched non-PPCM patients, with ICD implantation between 1996 and 2016 were identified. Device interrogation records were reviewed through 2018. Device therapy (shocks, antitachycardia pacing), device-related complications, and outcomes were analyzed.

**Results:**

Of 150 patients with PPCM, 20% (N = 30) underwent ICD implantation at median time from diagnosis of 7 months (IQR: 15 months) and left ventricular ejection fraction of 18% (IQR: 18%). Over 8 ± 6 years of ICD use (mean), 43% received appropriate device therapy (shock and/or antitachycardia pacing), similar to the NICM control group. Inappropriate device therapy occurred in 30% of patients with PPCM, most commonly due to supraventricular tachycardia. One-third of patients required at least one subsequent invasive ICD-related procedure other than generator replacement. After ICD implantation, 9 patients with PPCM (30%) had subsequent improvement of left ventricular ejection fraction to >50% and 4 of them had received appropriate ICD therapy. In comparison to the control group, there were no statistically significant differences in device therapy, despite longer ICD follow-up in the NICM control group (median 12 months vs 5 months, respectively, *P* < 0.05).

**Conclusions:**

In this cohort of patients with PPCM and ICD, rates of appropriate device therapy were high. Over long-term follow-up, rates of inappropriate shocks and device complications were also substantial in both PPCM and NICM cohorts.

Peripartum cardiomyopathy (PPCM) is a potentially life-threatening nonischemic cardiomyopathy characterized by left ventricular systolic dysfunction, defined by a left ventricular ejection fraction (LVEF) of <45%, diagnosed in the late antepartum or early postpartum period in women without known structural heart disease.^1^^,^^2^ Estimates of PPCM incidence are variable, ranging from 1 in 1,000 to 1 in 5,000 live births in the United States and Europe.^3^, ^4^, ^5^ Despite heightened recognition and diagnosis of PPCM since it was first described in the 1970s,^6^^,^^7^ many questions regarding optimal management remain unanswered.

Compared to other forms of nonischemic cardiomyopathy (NICM), PPCM is associated with higher likelihood of recovery of left ventricular function, with estimated recovery of left ventricular function approximately 72,^8^ with significant regional variation.^9^ As such, this creates confusion regarding the need for a primary prevention implantable cardioverter-defibrillator (ICD), and expert consensus recommends delay in ICD implantation to allow for recovery.^2^^,^^10^ Prior studies have shown that ICD implantation rates remain low for PPCM.^11^^,^^12^ Yet in high-risk populations of patients with PPCM, the risk of sudden death presumed secondary to ventricular tachyarrhythmias is high (approximately 25% of all deaths).^13^ In another study of a European cohort, 12% experienced ventricular arrhythmias.^14^ These data underscore the need for prevention of sudden death with ICD implantation in selected patients, while also allowing time for recovery of ventricular function. The use of wearable cardioverter-defibrillators (WCDs) has been proposed as a potential bridge, but its efficacy and translation to clinical outcomes in PPCM requires more study.^14^, ^15^, ^16^

There is a significant gap in our understanding of outcomes after ICD placement in patients with PPCM. Therefore, we sought to describe a cohort of patients with PPCM who underwent ICD placement. We compared long-term ICD-related outcomes in patients with PPCM vs an ethnicity-matched NICM control group.

## Methods

This was a retrospective cohort study at a large quaternary care center (University of Michigan Health, Ann Arbor, Michigan, USA) where specialized cardiovascular, electrophysiology, and high-risk obstetrical services are available. The study was approved by the Institutional Review Board of the University of Michigan.

### Patient identification

Patients with PPCM diagnosed between 1994 and 2016 were identified using ICD9 code 674.5x and the University of Michigan Electronic Medical Record Search Engine, which is a full-text search engine designed for information retrieval from narrative documents stored in electronic health records.^17^ All charts were manually reviewed in detail. To be included in the analysis, patients met the criteria for PPCM as defined by the 2010 European Society of Cardiology Working Group on Peripartum Cardiology ^1^ and only patients who underwent implantation of an ICD at our institution with available device interrogations were included.

For comparison, a one-to-one ethnicity-matched control group of female patients with non-PPCM dilated cardiomyopathy was identified using the same procedure as previously mentioned (University of Michigan Electronic Medical Record Search Engine and manual chart review) as well as an existing institutional electrophysiology database to ensure subjects had available device interrogation records.

### Data collection

Among patients who underwent ICD implantation, baseline clinical characteristics (eg, age, race, LVEF, NYHA functional class, laboratory data, medications used, comorbidities, number of pregnancies) and ICD-related variables (eg, indication for ICD implantation, type of ICD initially implanted, LVEF and New York Heart Association class at time of implantation) were manually collected from the medical record. LVEF at the time of device implantation was also categorized as per the American Society of Echocardiography classification system.^18^ ICD therapies, including both antitachycardia pacing (ATP) and ICD shocks were ascertained based on careful review of clinical documentation. Whenever available, device electrograms were also manually reviewed by the study team to ensure the accuracy of arrhythmia classification and the appropriateness of ICD therapies. Subsequent invasive device-related procedures and their indications were noted and tabulated. Among patients who initially underwent ICD implantation for the primary prevention of sudden cardiac death (SCD), device interrogation data were manually reviewed to determine whether initial ICD tachyarrhythmia programming was consistent with the 2015 HRS/EHRA/APHRS/SOLAECE expert consensus statement on optimal ICD programming intended to limit unnecessary shocks.^19^ Non-ICD-related clinical outcomes (LVEF recovery, left ventricular assist device implantation or heart transplant, and death) were manually collected from the medical record.

### Statistics

Clinical characteristics and outcomes were reported as frequencies with percentages, mean ± SD, or median (IQR). Univariate and bivariate comparisons were performed by nonparametric methods, including the Mann-Whitney U test and chi-square. ICD therapies were reported as the percentage of patients receiving no therapy, only appropriate therapy, any appropriate therapy, only inappropriate therapy, or both appropriate and inappropriate therapy. Due to censoring, survival comparison between the 2 cohorts was also performed using Kaplan-Meier survival estimates. Statistical analyses were performed using Stata Statistical Software: Release 18 (StataCorp LLC).

## Results

### Baseline clinical characteristics

Of 150 total patients with PPCM, 20% (n = 30) ultimately underwent ICD implantation and their baseline clinical characteristics are reported in Table 1. The majority of PPCM diagnoses were made in the postpartum period (85%) rather than antepartum. At the time of diagnosis, the mean LVEF was 23% ± 10% and mean NYHA functional class was 3.3 ± 1.0. Use of beta blockers and ACE inhibitors/angiotensin receptor blockers was common (90% and 87%, respectively), and bromocriptine use was uncommon (10%). The years of this study predated the use of sacubitril-valsartan and SGLT2-inhibitors.

**Table 1:** Baseline Clinical Characteristics of Patients With Peripartum Cardiomyopathy and ICD Placement (N = 30)

Age at diagnosis, y	26.6 ± 6.3
LVEF at diagnosis, %	23 ± 10
LVEF at longest follow-up, %	39 ± 21
Δ LVEF, percentage points	16 ± 22
NYHA class at diagnosis	3.3 ± 1.0
NYHA class at longest follow-up	2.0 ± 0.9
B-type natriuretic peptide at diagnosis, pg/mL	1,375 ± 1,428
B-type natriuretic peptide at longest follow-up, pg/mL	528 ± 925
Creatinine at diagnosis, mg/dL	0.9 ± 0.3
Creatinine at longest follow-up, mg/dL	1.2 ± 1.2
Number of pregnancies	2.7 ± 1.7
Postpartum diagnosis (of 27 known)	23 (85%)
Medications used	
Beta blocker	27 (90%)
ACE-inhibitor/angiotensin receptor blocker	26 (87%)
Loop diuretic	27 (90%)
Spironolactone	22 (73%)
Hydralazine	6 (20%)
Nitrates	8 (27%)
Digoxin	19 (63%)
Inotropes	14 (47%)
Bromocriptine	3 (10%)
Comorbidities	
Preexisting essential hypertension	5 (17%)
Gestational hypertension	2 (7%)
Preexisting diabetes mellitus	0 (0%)
Gestational diabetes mellitus	3 (10%)
Preeclampsia	5 (17%)
Thyroid disease	3 (10%)
Autoimmune disorder	1 (3%)
Depression/anxiety	5 (17%)

Values are mean ± SD or %.

LVEF = left ventricular ejection fraction.

### WCD utilization

Among the PPCM patients who underwent ICD implantation, 3 patients (10%) had worn a WCD as a bridge to a decision regarding permanent ICD implantation. An additional 3 wore a WCD as a bridge between ICD extraction and ICD reimplantation. These 6 patients did not receive any therapy from their WCDs.

### Clinical characteristics related to ICD implantation

Clinical characteristics and outcomes associated with ICD implantation for both the PPCM and NICM cohorts are reported in Table 2. Patients with NICM were older at the time of ICM implantation than patients with PPCM (*P* < 0.001). LVEF at the time of ICD implantation was lower in the PPCM group (median 18% vs 25%), but this was only marginally statistically significant (*P* = 0.07). The predominant indication for ICD implantation was the primary prevention of SCD (86%) in patients with PPCM, and this was slightly lower in the NICM cohort (67%, *P* = 0.09). Regarding device selection, patients with PPCM more frequently received a transvenous single chamber ICD (70%; n = 21), whereas more patients with NICM received a transvenous dual chamber ICD or cardiac resynchronization therapy-defibrillator (CRT-D). Among the 5 patients with PPCM whose initial ICD implantation took place after Food and Drug Administration approval and commercial availability of the subcutaneous ICD (S-ICD),^20^ 2 received an S-ICD. None of the NICM group received an S-ICD.

**Table 2:** Characteristics and Outcomes Among Patients With Peripartum Cardiomyopathy (PPCM) and Non-PPCM Control-Group With ICD Placement

	PPCM (n = 30)	NICM (n = 30)	*P* Value
Age at implantation, y	29 (11), [19-49]	57 (28) [15-85]	<0.001
Race/ethnicity			
Asian	2 (6.7%)	2 (6.7%)	a
Black	12 (40%)	12 (40%)	a
White	14 (46.7%)	14 (46.7%)	a
Other	2 (6.7%)	2 (6.7%)	a
Time from diagnosis to implantation, mo	7 (15), [0-148]	15 (56), [0-218]	0.48
LVEF at implantation	18 (18), [5-40]	25 (21), [10-75]	0.07
LVEF categories			
Normal (LVEF 54% and higher)	0	3 (12%)	0.12
Mild dysfunction (LVEF 41%-53%)	0	3 (12%)	0.12
Moderate dysfunction (LVEF 30%-40%)	5 (26%)	2 (8%)	0.10
Severe dysfunction (LVEF <30%)	14 (74%)	17 (68%)	0.67
LBBB at implantation, n (%) (of n = 20 known and n = 22 known, respectively)	1 (5%)	10 (45%)	<0.01
Primary prevention at initial implantation, n (%) (of n = 28 and n = 30 known, respectively)	24 (86%)	20 (67%)	0.09
Type of ICD at initial implantation			
Single-chamber ICD	21 (70%)	7 (23%)	<0.01
Dual-chamber ICD	5 (17%)	14 (47%)	0.01
Subcutaneous ICD	2 (7%)	0	0.15
CRT-D	2 (7%)	9 (30%)	0.02
Arrhythmias detected after implantation			
Atrial fibrillation	4 (13%)	18 (60%)	<0.01
SVT other than atrial fibrillation	11 (37%)	9 (30%)	0.58
VT not resulting in device therapy	12 (40%)	4 (13%)	0.02
VT resulting in device therapy	11 (37%)	8 (27%)	0.41
Polymorphic VT or VF	6 (20%)	5 (17%)	0.74
ICD Therapies			
ICD shocks			
No ICD shocks	16 (53%)	18 (60%)	0.60
Appropriate shock(s) only	6 (20%)	10 (33%)	0.24
Any appropriate shock(s)	11 (37%)	9 (30%)	0.58
Inappropriate shock(s) only	3 (10%)	3 (10%)	1.00
Both appropriate and inappropriate shock(s)	5 (17%)	1 (3%)	0.09
Antitachycardia pacing (ATP)			
No ATP	16 (57%)	16 (57%)	1.00
Any appropriate ATP	8 (30%)	11 (37%)	0.41
Appropriate ATP only	6 (21%)	10 (33%)	0.24
Inappropriate ATP only	4 (14%)	4 (14%)	1.00
Both appropriate and inappropriate ATP	2 (7%)	1 (3%)	0.56
Subsequent invasive ICD related procedures			
Any subsequent invasive ICD-related procedure (other than generator replacement)	10 (33%)	7 (23%)	0.39
Lead revision or implantation of new lead	4 (13%)	5 (17%)	0.72
ICD extraction due to infection	3 (10%)	1 (3%)	0.30
ICD extraction for failed defibrillation threshold testing or subclavian vein stenosis	1 (3.3%)	1 (3%)	1.00
Pocket revision	2 (7%)	2 (7%)	1.00
Upgrade to CRT-D	2 (7%)	5 (17%)	0.23
Clinical status at longest follow-up			
Alive, improvement of LVEF to >50%	9 (30%)	15 (50%)	0.11
Alive, no recovery or partial recovery of LVEF to ≤50%	8 (27%)	10 (33%)	0.58
Alive with left ventricular assist device	1 (3%)	3 (10%)	0.30
Alive with heart transplant	4 (13%)	3 (10%)	0.69
Death	8 (27%)	5 (17%)	0.35
Duration of ICD use to date, y (time from implant to last charting), median (IQR), [range]	5 (7), [0-20]	12 (10), [1-20]	0.00034

CRT-D = cardiac resynchronization therapy with defibrillator; ICD = implantable cardioverter-defibrillator; LBBB = left bundle branch block; LVEF = left ventricular ejection fraction; NA = not applicable; SVT = supraventricular tachycardia; VF = ventricular fibrillation; VT = ventricular tachycardia.

a PPCM and NICM groups were one-to-one matched on race/ethnicity.

### ICD therapies

Of the patients with PPCM, the total mean duration of follow-up was 11 ± 17 years. Over a mean duration of ICD use of 8 ± 6 years, 37% (n = 11) patients received at least 1 appropriate ICD shock for VT (20%; n = 6) and/or polymorphic VT/ventricular fibrillation (20%; n = 6). Inappropriate shocks occurred related to supraventricular tachycardia (SVT) in 8 patients. One of these patients also received an inappropriate shock for T-wave oversensing. Among patients with a transvenous ICD system, ATP delivery was also common with 8 patients (29%) receiving appropriate ATP for ventricular arrhythmias and 6 patients (36%) receiving inappropriate ATP for supraventricular rhythms. A total of 13 patients (43%) received appropriate device therapy (ATP and/or shock).

Of the patients with NICM, high rates of appropriate shocks (n = 9, 30%) and appropriate ATP (n = 11, 37%) for ventricular arrhythmias also occurred. Patients with NICM were more likely to have atrial fibrillation (60% vs 13%, *P* < 0.01), but did not have statistically higher rates of inappropriate ICD therapies.

### ICD tachyarrhythmia programming

Among patients with PPCM known to have had implantation of ICD for the primary prevention of SCD, a minority (38%) had initial ICD tachyarrhythmia detection programming consistent with current consensus guidelines intended to limit unnecessary shocks.^19^ It should be noted that all but one of these patients received their initial implant before the publication of these guidelines in 2015. There was no statistically significant difference between the rate of appropriate and inappropriate shocks between those whose devices were consistent with national guidelines and those who were not (*P* = 0.512).

### Subsequent device-related procedures

One-third (n = 10; 33%) of patients with PPCM required at least 1 subsequent invasive ICD-related procedure other than generator replacement. The most common type of procedure was device extraction, 3 of which were for device-related infections and 1 for noninfectious reasons (failed defibrillation threshold testing despite lead revision). Four patients (13%) required lead revision or new lead insertion and 2 patients (7%) required upgrade to a CRT-D when they developed an indication for CRT after their initial ICD implantation. No device-related infections occurred related to the 2 subcutaneous-ICDs. In the NICM control group, rates of subsequent invasive procedures were similar; however, this group also had a longer duration of follow-up (Table 2).

### Long-term clinical outcomes

Among patients with PPCM, at long-term follow up (mean 11 ± 17 years), nearly one-third who underwent ICD implantation (n = 9; 30%) were alive and had experienced improvement of LVEF to >50%. Five of these patients (55%) with LVEF recovery had never received any appropriate ICD therapies for ventricular arrhythmias. Twelve patients (40%) required advanced heart failure therapies, which included 6 patients with heart transplantation (4 had prior left ventricular assist device) and 6 patients with destination left ventricular assist device. Death occurred in 8 (27%) of patients. Although there were no statistically significant differences in these outcomes by univariate analyses, a log-rank test of equality of the survival function demonstrated that patients with PPCM had shorter time to death over the duration of follow-up than those with NICM (*P* = 0.04).

## Discussion

The main findings from this article are that among patients with PPCM 1) 20% underwent ICD implantation, 2) the rate of appropriate therapy was high (43% over a median follow-up of 5 months), 3) one-third of patients received inappropriate device therapy, and 4) one-third of patients developed complications requiring an invasive ICD-related procedure. In comparison to the NICM control group, rates of appropriate/inappropriate therapy and subsequent invasive procedures were similar.

As the incidence of PPCM is increasingly recognized, the electrophysiologic consequences, such as SCD and other arrhythmias, are important considerations in this population. The vast majority of patients with PPCM were on the guideline-directed heart failure therapies available at that time, and all patients met guideline criteria for ICD implantation. Importantly, 43% of the patients with PPCM in this study received at least 1 appropriate ICD therapy (shock and/or ATP), similar to race/ethnicity matched women with NICM. Inappropriate shocks occurred in 27% of patients with PPCM, most commonly in the setting of SVT. While the rate of inappropriate shocks is lower following advancements in ICD programming (1.5% to 5%),^21^, ^22^, ^23^ historical rates preceding many of these device changes were around 14 to 48% and often occurred in the setting of SVT.^21^^,^^24^, ^25^, ^26^ Updated programming algorithms with better identification of SVT algorithms has since led to this reduction in inappropriate device therapy.^23^^,^^27^

ICD-related outcomes in patients with PPCM and NICM were similar. Interestingly, both groups had relatively high rates of appropriate device therapy for ventricular arrhythmias. Subsequent invasive ICD-related procedures were similar in both groups, albeit with a longer duration of follow-up in the NICM group. As expected, given the difference in age and comorbidities, those undergoing ICD placement for NICM were more likely to have an underlying LBBB and underwent placement of a CRT-D or dual chamber ICD as opposed to the single chamber ICD for those with PPCM. Furthermore, patients with NICM were more likely to have atrial fibrillation detected on device interrogations following implantation which is more common amongst those with older age and has better discrimination for detection in those with implantation of an atrial lead.

At the time of the study, the use of WCDs was low, with 10% utilizing a WCD prior to ICD implantation. Studies have demonstrated that WCD use is reasonable to consider for 3 to 6 months following diagnosis of PPCM, as recovery of LVEF is most common during this timeframe.^2^^,^^8^^,^^14^^,^^16^^,^^28^ Arrhythmias requiring device discharge have been reported as less frequent in patients with PPCM in comparison to those with NICM not associated with pregnancy.^15^ In contrast, our findings suggest high rates of device therapy (shock and/or ATP). Delayed recovery after 2 years or more is not uncommon in PPCM^2^^,^^11^ and premature implantation should be avoided; however, risk of arrhythmia even after recovery is not well-defined. While the most recent 2022 AHA/ACC/HFSA heart failure guidelines do not mention the use of WCDs in PPCM or for other types of cardiomyopathies, the 2017 AHA/ACC/HRS Guidelines for Management of Patients with Ventricular Arrhythmias do address the notion of WCDs as a bridge while awaiting potential recovery.^29^ Recovery of LVEF and, therefore, timing of ICD implantation is an important consideration given the relatively young age of this patient population and the associated potential long-term risks of device complications. Significantly, one-third of women required subsequent invasive ICD-related procedures other than routine generator replacement (ie, pocket revision, lead revision, extraction, upgrade to CRT-D).

Nearly a third of patients with PPCM had recovery of LV systolic function (LVEF >50%) after ICD placement, despite the fact that the mean time to implantation was relatively late (average of 26 months after diagnosis). Among the 9 patients who had recovery, 5 (55%) had not received any therapy for ventricular tachyarrhythmias and may not require lifelong ICD therapy, though the long-term risk of SCD in this subpopulation is not known. In patients with a history of a reduced ejection fraction (not related to PPCM) with subsequent recovery, retrospective studies demonstrated up to 36% of patients with a recovered ejection fraction will receive appropriate ICD shocks following recovery; however, rates are typically lower among those with NICM.^30^, ^31^, ^32^ Additional study is needed to address whether patients with recovered PPCM should undergo generator replacement or device reimplantation if complications (eg, infection) arise.

Only 38% of patients with PPCM had ICD programming compatible with the recommended tachycardia detection programming in the 2015 HRS guidelines, though all but 1 patient had their initial implant before these guidelines were published. The absence of guideline-compatible programming was not associated with ICD shocks in this relatively small sample. Prior studies of permissive ICD programming (higher VT/VF zone cutoffs; longer detection times) in primary prevention patients have included NICM patients but not specifically PPCM patients.^19^^,^^21^^,^^23^, ^24^, ^25^^,^^33^

The S-ICD was approved by the Food and Drug Administration in 2012. Of the 5 patients with PPCM whose initial implant occurred in the era of commercial availability of the S-ICD, 2 received the S-ICD. The S-ICD may play an important role in PPCM patients given their relatively young age at the time of initial implant and the potential to avoid long-term problems related to endovascular access or need for device extraction. On the other hand, nearly one-third of the patients in our sample received appropriate ATP therapies, which has been shown to reduce dependency on ICD shocks and which currently available S-ICD systems cannot offer.^19^^,^^24^^,^^25^^,^^29^ Furthermore, 7% of our sample required an eventual upgrade to a CRT system, the capabilities of which are not possible with the current S-ICD systems. With the Food and Drug Administration approval of the extravascular ICD (EV-ICD) in 2023, which allows for ATP delivery, future studies are needed to understand the utilization and outcomes in patients with PPCM. Our study was not designed to determine whether S-ICD is an optimal ICD choice in young PPCM patients without a pacing indication, and this remains an important area of future study. The majority of our patients had transvenous ICD systems; while several benefited from ATP capability, risks of infection and inappropriate shocks were not insignificant. Additionally, while most PPCM patients do not require pacing, future modular systems utilizing leadless pacemakers can benefit patients who receive an S-ICD at the time of initial implantation but who later develop a compelling need for pacing.

Our study strengths include the well-characterized cohort of PPCM patients and a NICM control group with highly granular data including echocardiographic results, long-term follow-up, and detailed ICD interrogation reports. This is the largest study examining detailed ICD-related outcomes in PPCM patients to date, and it identifies future topics for exploration in this population. Patients in this study had long duration of follow-up, providing important information on LV systolic function recovery and overall need for device therapies. Lastly, patients included in our study had routine and consistent device clinic follow-up, limiting the possibility of under-diagnosing events or ICD therapies and ensuring that follow-up device procedures were included.

There are also several limitations to this study. First, this was a descriptive, observational study. Our analysis was limited by small sample sizes and availability of data from chart review. The control group was matched based on race/ethnicity; however, other confounders such as age, comorbidities, and prior pregnancy history would be important to consider. Our findings may be useful for counseling PPCM patients about ICD implantation, but timing of ICD implantation and selection of device (ICD vs S-ICD) require further study. Second, at the time of this study, sacubitril-valsartan and SGLT2-inhibitors were not available, and are now associated with substantial benefit for patients heart failure with reduced ejection fraction.^34^ Third, referral bias occurred at this single, large quaternary care center since patients are referred here for management of complications not routinely managed in community settings. Finally, a few device electrograms could not be reviewed due to events or therapies occurring before records were systematically uploaded, or in patients arriving from another hospital, potentially limiting full data analysis.

This study highlights the need for future research regarding device therapy in patients with PPCM. Areas of future study include appropriate timing of device placement given the substantial, and often delayed, recovery of LV systolic function (Central Illustration). The fact that many patients still benefited from therapy despite subsequent ejection fraction recovery will require further clarification. Similarly, management strategies for patients with myocardial recovery, choice of long-term medications, device therapies, considerations for future pregnancies, and serial follow-up must all be considered and better identified. Choice of device (WCD duration, S-ICD, transvenous ICD) in this young population at risk for long-term re-intervention and possible complications will also need to be better understood, as well as approach to removal of devices that may no longer provide substantial benefit.

**Central Illustration:**
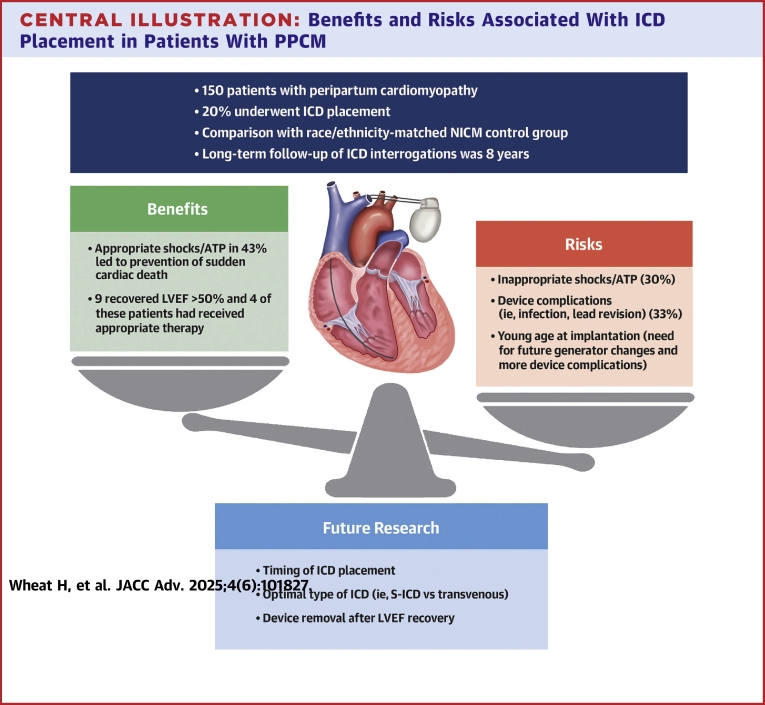
**Benefits and Risks Associated With ICD Placement in Patients With PPCM** Among young women with PPCM (peripartum cardiomyopathy), risks of inappropriate shocks and subsequent invasive procedures should be considered; however, the substantial benefit of appropriate device therapy for life-threatening arrhythmias should tip the scales in properly selected patients. ATP = antitachycardia pacing; ICD = implantable cardioverter-defibrillator; LVEF = left ventricular ejection fraction; S-ICD = subcutaneous ICD.

## Conclusions

Our study demonstrated that a substantial proportion of individuals who undergo ICD implantation for PPCM subsequently receive appropriate device therapy for ventricular arrhythmias at rates similar to other forms of NICM. A proportion of patients subsequently had recovery of LV systolic function after ICD implantation, suggesting further research could answer questions about appropriate timing, potential device extraction, and optimal type of device placement. Overall, despite long-term risks of device complications and inappropriate shock therapy, the high rates of appropriate device therapy highlights the importance of ICD use in appropriately-selected patients with PPCM.

Perspectives **COMPETENCY IN MEDICAL KNOWLEDGE:** Although many patients with PPCM have subsequent recovery of LV systolic function, those who eventually undergo ICD implantation experience appropriate ICD therapies at relatively high rates, at the cost of inappropriate ICD therapies and possible need for subsequent device-related procedures other than routine generator replacements.

**TRANSLATIONAL OUTLOOK:** Further studies are needed to determine the optimal timing of ICD implantation and optimal device selection in PPCM patients who meet criteria for ICD implantation.

## Funding support and author disclosures

The authors have reported that they have no relationships relevant to the contents of this paper to disclose.
